# Epidemiological characteristics and natural history of porphyria – a twenty-year population-based analysis in Taiwan

**DOI:** 10.1186/s13023-025-04110-7

**Published:** 2025-11-17

**Authors:** Wei-Chih Wu, Yun-Hsuan Yeh, Chia-Chun Li, Yao-Tseng Wang, Yin-Fan Chang, Chin-Sung Chang, Ru-Hsueh Wang, Jiaan-Der Wang, Hung-Chou Kuo, Chung-Hwan Chen, Chih-Hsing Wu

**Affiliations:** 1 Department of Family Medicine, National Cheng Kung University Hospital, College of Medicine, National Cheng Kung University, No. 138, Shengli Rd., North Dist, Tainan City, 704302 Taiwan; 2 Division of Hematology and Oncology, Department of Pediatrics, Ditmanson Medical Foundation Chiayi Christian Hospital, Chiayi, Taiwan; 3 Institute of Allied Health Sciences, College of Medicine, National Cheng Kung University, Tainan, Taiwan; 4 Center for Rare Disease and Hemophilia, Taichung Veterans General Hospital, Taichung City, Taiwan; 5 Department of Neurology, Chang Gung Memorial Hospital Linkou Medical Centre and College of Medicine, Chang Gung University, Taoyuan, Taiwan; 6 Department of Orthopedics, Kaohsiung Medical University Hospital, Kaohsiung Medical University, No. 100, Shiquan 1st Rd., Sanmin Dist, Kaohsiung City, 807378 Taiwan; 7 Orthopedic Research Center, College of Medicine, Kaohsiung Medical University, Kaohsiung, Taiwan; 8 Department of Family Medicine, College of Medicine, National Cheng Kung University, Tainan, Taiwan; 9 Institute of Gerontology, College of Medicine, National Cheng Kung University, Tainan, Taiwan

**Keywords:** Rare diseases, Mortality, Health care utilization, Specialties, Early diagnosis

## Abstract

**Background:**

Porphyrias are a group of rare, genetic disorders of heme biosynthesis in which reduced activity of any of the eight pathway enzymes leads to accumulation of intermediates and distinct clinical syndromes. These disorders are heterogeneous—not a single polymorphic condition—and are associated with impaired quality of life and increased mortality. Global epidemiological data, particularly from Asia, remain limited. This study analyzes population-based data from Taiwan to provide real-world evidence.

**Methods:**

This study utilized the Taiwan National Health Insurance Research Database (~ 23 million population), which is linked with the Cause of Death Database and the Registry for National Health Insurance Catastrophic Illness Card database. Cases were validated through the rare disease registry of the Health Promotion Administration. The annual case numbers (2002–2022) were analyzed, with follow-up data on survival and causes of death. Outpatient and inpatient visits by specialty (2001–2022) and the average annual number of medical visits per person (2001–2022), including outpatient, inpatient and emergency visits, were assessed.

**Results:**

Among 126 patients with porphyria, 77.0% were female, with a mean age at diagnosis of 35.12 years. The annual incidence ranges from 0.04 to 0.62 per million, with a prevalence of 5.15 per million in 2022. The neurology, family medicine, and otolaryngology departments accounted for the most outpatient visits, whereas hospitalizations were common in the pediatrics, neurology, and gastroenterology departments. The mortality rate was 1543.4 per 100,000 people, and healthcare utilization was significantly 2-fold and above than that in the general population.

**Conclusions:**

The incidence and prevalence of porphyria in Taiwan are lower than those reported in previous studies. The high mortality rate and diverse clinical manifestations emphasize the need for early diagnosis and timely management to reduce the disease burden.

**Supplementary Information:**

The online version contains supplementary material available at 10.1186/s13023-025-04110-7.

## Background

Porphyrias are a group of rare, defined as a prevalence rate < 1/10,000, and metabolic disorders of heme biosynthesis. Each specific porphyria arises from reduced activity of one of the eight enzymes in the pathway, leading to accumulation of upstream intermediates and characteristic biochemical signatures in plasma, erythrocytes, urine, or feces. Clinically, porphyrias are commonly categorized by the principal site of overproduction/accumulation (hepatic vs. erythropoietic) and by presentation—acute neurovisceral attacks (the acute hepatic porphyria) and/or cutaneous photosensitivity [1, 2].

Acute hepatic porphyria (AHP) comprises four phenotypes: acute intermittent porphyria (AIP), hereditary coproporphyria, variegate porphyria, and 5-aminolevulinic acid dehydratase deficiency porphyria [3, 4]. AHP has been reported to be associated with comorbidities such as chronic pain, primary liver cancer, kidney cancer, chronic kidney disease, hypertension, venous thrombosis disease, hemorrhagic stroke, and psychiatric disease [5–7]. These conditions can result in a reduced quality of life and increased mortality. The overall mortality rate in patients with AHP is higher than that in the general population [5].

A rare disease (RD) affects a small portion of the population, and the criteria for defining RDs vary among countries. RDs are classified as conditions with fewer than 200,000 cases nationwide in accordance with the Rare Disease Act of 2002; in Europe, RDs have fewer than 1 case in 2,000 individuals according to the European Commission. In 2000, Taiwan implemented the “Rare Disease and Orphan Drug Act”, which defines RDs as those with a prevalence lower than the standard set by the central government or with special circumstances. These diseases are subject to review by the “Review Committee for Rare Diseases and Orphan Drugs” and are officially announced by the central government. The incidence rate for RDs is lower than 1 in 10,000.

In European countries, the incidence and prevalence of symptomatic porphyria range from 0.33 per million individuals and 18.3 per million individuals, respectively [8]. A population-based study in Germany reported that the prevalence of AHP was 79.8 cases per million individuals [9]. An Argentinean study reported that the prevalence of AIP and VP was approximately 9.67 per million individuals [10]. However, Asian data on the epidemiology of porphyria are very limited [4, 11, 12]. A study conducted by a local bank in Hebei Province, China, reported an annual incidence of AIP ranging from 0.03 to 0.05 cases per million people [11]. To the best of knowledge, there is no nationwide study report on porphyria in Asia.

Diagnosing porphyria is complex and typically integrates clinical assessment with laboratory confirmation. The diagnostic approach used at a tertiary medical center in Taiwan—covering: clinical symptoms evaluation; biochemical confirmation with urinary (or plasma) ALA/PBG, supported by porphyrin profiling and plasma fluorescence; enzyme assays when available; genetic testing when feasible; genetic counseling and patient education; targeted neurologic assessment and serum sodium monitoring during attacks; and longitudinal follow-up (≥ 1 year) with periodic urinary ALA and/or PBG to establish inter-attack baselines and document attack patterns [13]. For follow-up, the same institution’s supervision framework emphasizes structured clinic follow-up with education and trigger avoidance; periodic urinary ALA/PBG between attacks to maintain individual baselines and document attacks; EHR-based medication alerts to prevent porphyrogenic prescribing; predefined escalation for recurrent attacks (e.g., hormonal therapy, givosiran for clinically/biochemically confirmed AIP with recurrent attacks, and prophylactic heme when attacks are frequent); and annual hepatic/renal surveillance [13]. 

Owing to its rarity, porphyria may be underdiagnosed, resulting in delayed management. Therefore, physicians in relevant medical specialties should keep in mind that patients seeking medical consultation for porphyria exhibit early-stage symptoms. Real-world evidence is valuable for reaching epidemiology in rare disease research [14]. This study used mega data from Taiwan population-based database and represents comprehensive information of epidemiology study of patients with porphyria.

## Method

### Data source

This study relied on information extracted from the Taiwan National Health Insurance Research Database (NHIRD), a population-based database of health insurance claims covering nearly all residents of Taiwan. The Taiwan National Health Insurance (NHI) program was initiated in 1995, and as of the 2021–2022 period, it provided coverage to 99.9% of Taiwan’s population, totaling 23,876,603 individuals, as documented in the 2021–2022 National Health Insurance Annual Report. The database, established by the National Health Insurance Administration, has collected medical claims population-based data over the years, providing comprehensive details on outpatient and inpatient care expenses, disease diagnoses, and prescriptions [15].

The NHIRD, aided by the Health and Welfare Data Science Center, encompasses both outpatient and inpatient expenditures. It is linked to the Cause of Death Database to determine mortality status and causes of death, as well as to the Registry for NHI Catastrophic Illness Card database to identify patients with rare diseases. The research received ethical approval from the Institutional Review Board at National Cheng Kung University Hospital under reference number IRB No. B-ER-109-416 and is also registered with ClinicalTrials.gov (No. NCT05367115).

### Study cohort identification and follow-up

Porphyria patients in Taiwan could be validated by receiving catastrophic illness certification through the RD registry of the Health Promotion Administration. Before the Health Promotion Administration, the Ministry of Health and Welfare in Taiwan issued a certification card for porphyria. All conditions must be fully satisfied in the patient’s clinical history (such as age at disease onset, acute neurovisceral symptoms, skin symptoms, first attack or not, frequency of acute attacks), family history, and laboratory examinations, including tests for elevated porphobilinogen (PBG), delta-aminolevulinic acid (ALA), and porphyrin levels in urine/plasma; plasma fluorescence emission peak; and porphobilinogen deaminase. The Watson‒Schwartz test is selective. Recently, a formal report of genetic mutations and a clinical diagnosis of the porphyria subtype are necessary [13, 16].

Therefore, obtaining NHI catastrophic illness certification through the RD registry enhances the validity of the diagnosis for patients with porphyria. To prevent the inclusion of misdiagnosed patients, we initially identified individuals from the catastrophic illness certification card database and subsequently acquired relevant health-related data from the NHIRD. The index date of the cohort included in this study is the validation date in the catastrophic illness database since 2002. To examine new and cumulative case trends of porphyria identified in the cohort of this database, we calculate the annual case number of patients in Taiwan between 2002 and 2022. For all participants’ deaths, information on survival from the index date until 2022 was obtained from the Cause of Death Database, and the underlying cause of death was defined by the International Classification of Diseases 10th Revision (ICD-10) code.

Between 2001 and 2022, the total number of outpatient and inpatient visits by patients with porphyria for each medical specialty was calculated from the NHIRD. The follow-up years are calculated from the index date until the end of 2022 or the death date. The main diagnosis of medical seeking was analyzed by the International Classification of Diseases 9th Revision (ICD-9) and the ICD-10 codes. The average annual number of medical visits per person from 2001 to 2022, including outpatient visits, emergency department visits, and hospital admissions, was measured to evaluate healthcare utilization in patients with porphyria.

### Covariates

Patient characteristics at baseline included sex, age, follow-up year, survival status, cause of death, mortality rate, incidence and prevalence of porphyria, number of outpatient and inpatient visits by specialty, main diagnosis of outpatient visit and hospital admission, and number of medical visits, including outpatient visits, emergency visits and hospital admissions. This study utilized both the ICD-9 and the ICD-10 systems. Since the data spanned 22 years, diagnostic records initially used the ICD-9 code before 2015 and gradually transitioned to the ICD-10 code starting in 2016. Therefore, it was necessary to consider both coding systems.

### Statistical analysis

The prevalence per 1,000,000 persons was defined as the number of surviving porphyria patients with a catastrophic illness card divided by the mid-year population. The incidence rate (per 1,000,000 persons) was calculated by dividing the number of newly approved Catastrophic Illness Card applications among porphyria patients by the mid-year population during the same period. The average annual number of medical visits per person, including outpatient visits, emergency department visits, and inpatient admissions, was determined by dividing the total number of medical visits by the total number of patients with porphyria with a catastrophic illness card each year. All the data were analyzed via SAS software^®^, version 9.4 (SAS Institute Inc., Cary, NC, USA).

## Results

From 2002 to 2022, the average resident population was approximately 23 million. During this period, 128 individuals were diagnosed with porphyria. After the sex registry through 2022 was reviewed and two patients of unknown sex were excluded, 126 patients with porphyria were included in the final analysis.

**Fig. 1:**
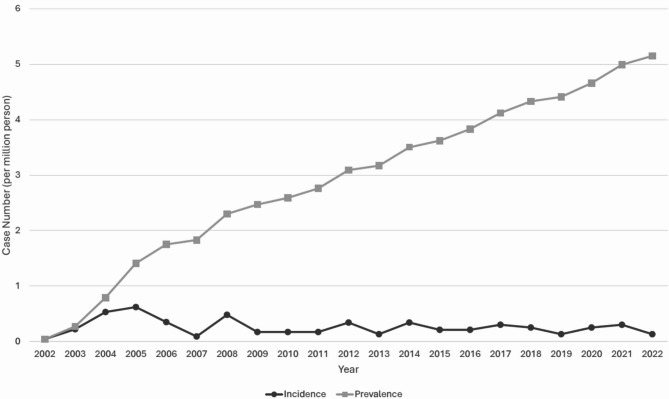
Trends in the incidence and prevalence of porphyria in patients in Taiwan between 2002 and 2022

As shown in Table 1, porphyria was more common in females (77.0%). The mean age at diagnosis was 35.12 years (SD: 13.56, range: 0–84), with a mean follow-up duration of 10.32 years (SD: 5.97, range: 0–20). During follow-up, eight patients died in 6 different year (2009, 2012, 2014, 2017, 2018, and 2022), resulting in a mortality rate of 1543.4 per 100,000 in average (Supplemental Table S1). As illustrated in Fig. 1, the annual incidence consistently low, from 0.04 to 0.62 per 1,000,000 persons, whereas the prevalence increased from 0.04 per million in 2002 to 5.15 per million in 2022 (Supplemental Table S1). On the basis of data from the Cause of Death Database, the underlying causes of death for eight patients were identified via the following ICD-10 codes: sepsis (A419), liver cancer (C229), dementia (F03), nontraumatic intracranial hemorrhage (I629), cerebrovascular disease (I679), pneumonia (J189), vascular disorders of the intestine (K559), and cellulitis and acute lymphadenitis (L039) (Supplemental Table S2).

**Table 1:** Baseline characteristics of the patients with porphyria included in this study between 2002 and 2022

	Total, No. (%)
Sex	126 (100.0)
Male	29 (23.0)
Female	97 (77.0)
Age at validation, mean (SD), y	35.12 (13.56)
Follow-up year, mean (SD), y	10.32 (5.97)
Average age of death, mean (SD), y	62.38 (18.93)
Survival status	
Survival	118 (93.7)
Death	8(6.3)
Mortality rate (2002–2022) †	1543.4 (per 100,000)

Data are n (%) or mean (SD)

^†^Years with zero deaths are excluded from the calculation

As shown in Table 2, between 2001 and 2022, the five outpatient departments most frequently visited by patients with porphyria were neurology (10448 visits), family medicine (6779 visits), otolaryngology (5493 visits), internal medicine (5046 visits), and obstetrics and gynecology (3886 visits). Table 3 presents the primary outpatient diagnoses for patients with porphyria between 2001 and 2022, which are based on the ICD-9 and ICD-10 codes (Supplemental Table S2). According to the ICD-9 coding, the most common diagnoses were disorders of porphyrin metabolism (7918 times), acute upper respiratory infections at unspecified sites (2289 times), acute sinusitis, unspecified infections (1048 times), acute tonsillitis (620 times), and acute bronchitis (615 times). The most frequent diagnoses on the basis of ICD-10 codes were acute intermittent (hepatic) porphyria (3978 times), unspecified porphyria (3668 times), hereditary erythropoietic porphyria (1025 times), other porphyria (640 times), and acute upper respiratory infections at unspecified sites (550 times). The average number of outpatient visits per patient per year ranged from 15.99 to 35.54 (Supplemental Table S3).

**Table 2:** Common medical departments of outpatient visits and inpatient admissions for 126 porphyria patients between 2001 and 2022 †

Outpatient department	No.	Inpatient department	No.
Neurology	10,448	Pediatrics	904
Family Medicine/General Practitioner	6779	Neurology	618
Otolaryngology	5493	Gastroenterology	440
Internal Medicine	5046	Oncology	263
Obstetrics & Gynecology	3886	Nephrology	195
Gastroenterology	3345	Internal Medicine	170
Psychiatry	3175	Psychiatry	167
Dermatology	3035	Rheumatology	93
Pediatrics	2972	Obstetrics & Gynecology	85
Ophthalmology	2813	Surgery	62

^†^ Healthcare utilization data were retrieved for one year prior to diagnosis (2001)

**Table 3:** The main diagnosis of outpatient visits for 126 patients with porphyria between 2001 and 2022 †

Outpatient (ICD-9)	No.	Outpatient (ICD-10)	No.
Disorders of porphyrin metabolism	7918	Acute intermittent (hepatic) porphyria	3978
Acute upper respiratory infections of unspecified site	2289	Unspecified porphyria	3668
Acute sinusitis, unspecified	1048	Hereditary erythropoietic porphyria	1025
Acute tonsillitis	620	Other porphyria	640
Acute bronchitis	615	Acute upper respiratory infections of unspecified site	550
Acute nasopharyngitis (common cold)	587	End stage renal disease	415
Essential hypertension, unspecified	570	Dysthymic disorder	321
Unspecified contact dermatitis, unspecified cause	451	Essential hypertension, unspecified	247
Other acne	449	Porphyria cutanea tarda	242
Acute pharyngitis	448	Kidney transplant status	227

Since 2016, diagnostic codes in Taiwan have gradually transitioned from the International Classification of Disease-9 (ICD-9) to the International Classification of Disease-10 (ICD-10)

^†^ Healthcare utilization data were retrieved for one year prior to diagnosis (2001)

As shown in Table 2, the most frequently visited inpatient departments for patients with porphyria between 2001 and 2022 were pediatrics (904 admissions), neurology (618 admissions), gastroenterology (440 admissions), oncology (263 admissions), and nephrology (195 admissions). Table 4 reports the main diagnoses for inpatient admissions during this period, which are based on the ICD-9 and ICD-10 codes. According to the ICD-9 coding, the most common diagnoses were disorders of porphyrin metabolism (1,071 times), paralytic ileus (25 times), paranoid-type schizophrenic disorder with acute exacerbation (21 times), other and unspecified noninfectious gastroenteritis and colitis (20 times), and unspecified diffuse connective tissue disease (20 times). On the basis of ICD-10 coding, the most frequent diagnoses were acute intermittent (hepatic) porphyria (664 times), unspecified porphyria (395 times), other porphyria (120 times), paranoid schizophrenia (41 times), and sepsis, unspecified organism (12 times). The average annual hospitalization rate per patient ranged from 2.06 to 6.58, with the average length of stay in an acute care bed varying from 13.53 to 55.31 days per year (Supplemental Table S3). Additionally, the average number of emergency visits per patient per year ranged from 2.85 to 9.11 (Supplemental Table S3).

**Table 4:** The main diagnosis of inpatient admissions for 126 porphyria patients between 2001 and 2022 ^†^

Inpatient (ICD-9)	No.	Inpatient (ICD-10)	No.
Disorders of porphyrin metabolism	1071	Acute intermittent (hepatic) porphyria	664
Paralytic ileus	25	Unspecified porphyria	395
Schizophrenic disorders, paranoid type, chronic with acute exacerbation	21	Other porphyria	120
Other and unspecified noninfectious gastroenteritis and colitis	20	Paranoid schizophrenia	41
Unspecified diffuse connective tissue disease	20	Sepsis, unspecified organism	12
Abdominal pain, unspecified site	18	Urinary tract infection, site not specified	11
Other specified types of schizophrenia, unspecified	15	Unspecified abdominal pain	11
Acute bronchitis	10	Iron deficiency anemia secondary to blood loss (chronic)	9
Urinary tract infection, site not specified	10	Pneumonia, unspecified organism	8
Intestinal or peritoneal adhesions with obstruction(postoperative)(postinfection)	9	Antiphospholipid syndrome	7

Since 2016, diagnostic codes in Taiwan have gradually transitioned from the International Classification of Disease-9 (ICD-9) to the International Classification of Disease-10 (ICD-10)

^†^ Healthcare utilization data were retrieved for one year prior to diagnosis (2001)

## Discussion

This study represents the first territory-wide, population-based cohort with complete population coverage and a 20-year follow-up, providing a more comprehensive investigation of the porphyria epidemiology than previous studies [4, 9]. This study included only rigorously diagnosed patients who met the strict criteria for the issuance of a catastrophic illness card. Therefore, this study offers valuable insights into the epidemiology and natural history of porphyria from a population-level perspective. Patients with porphyria exhibited significantly greater healthcare utilization, including outpatient visits, emergency department visits, and hospital admissions, with rates several times greater than those of the general population. This substantial disease burden not only diminishes quality of life but also places significant strain on the healthcare system. Early diagnosis is a critical initial step in improving clinical outcomes and reducing this burden [17].

In 2013, Taiwan introduced porphyria diagnostic kits for hereditary forms, sponsored by the Taiwan Foundation for Rare Disorders and developed by the Chinese Foundation of Health. Registration in the rare disease registry required elevated PBG and ALA levels, plasma fluorescence scans, and porphobilinogen deaminase tests. These kits focused on AHP, especially AIP, though plasma scans had limited accuracy for cutaneous or variegate types. To improve reliability, the Kuo team at Linkou Chang Gung University redeveloped a new detection method [18]. A major advancement occurred in December 2020, when the Rare Disease and Drug Review Committee approved mandatory genetic testing as a diagnostic criterion, significantly improving the accuracy of hereditary porphyria diagnosis. In Taiwan, obtaining certification for porphyria requires meeting stringent diagnostic criteria to qualify for both RD certification and the Catastrophic Illness Card. Strict diagnostic criteria ensure the accuracy of the porphyria diagnosis. Lissing et al. studied porphyria patients in Sweden, all of whom had confirmed pathogenic mutations [5]. In a study conducted in the USA, 108 porphyria patients were included on the basis of typical symptoms and biochemistry test results, with 105 of these cases confirmed by genetic mutation [19]. A separate study in Japan revealed that porphyria diagnoses were based primarily on clinical judgment, considering symptoms and laboratory data, as genetic testing was not covered by national insurance at the time [4]. However, the strict diagnostic criteria required for Catastrophic Illness certification may delay diagnosis and lead to fewer confirmed cases. A significant proportion of outpatient visits were attributed primarily to acute upper respiratory infections, skin conditions like contact dermatitis and acne and hypertension (Table 3). The nonspecific nature of these symptoms, combined with the relatively young age of many patients, makes diagnosing porphyria even more challenging. These factors may have contributed to the lower annual incidence and prevalence rates of porphyria in this study than in European countries [8], Germany [9] and Argentina [10].

Because patients with porphyria present across multiple specialties, raising awareness of potential porphyria cases among physicians in commonly visited outpatient specialties—such as neurology, family medicine, otolaryngology, internal medicine, and obstetrics and gynecology—remains a challenge (Table 2). A German cohort study reported that 39.3% of all visits made by AHP patients were to general practitioners, underscoring the importance of physician awareness in outpatient settings [9]. Notably, most hospitalized porphyria patients are diagnosed with porphyria-related ICD-9 and ICD-10 codes (Table 4). The provision of a Catastrophic Illness Card, which offers medical expense reductions, may increase the accuracy of primary diagnoses for inpatient admissions among patients with porphyria. Pediatrics was the most frequently recorded inpatient department (Table 2). This likely reflects the early onset of porphyria symptoms in adolescence and limited awareness among patients and clinicians, leading to frequent acute attacks and hospitalizations. Other major inpatient departments—neurology, gastroenterology, oncology, nephrology, internal medicine, and rheumatology—are presumed to reflect adult care, as pediatric patients in Taiwan with conditions relevant to these specialties are typically admitted under general pediatrics. This is likely due to the lack of distinct categorization for pediatric subspecialties (e.g., pediatric nephrology or pediatric neurology) in hospital records. As shown in Table 2, pediatric admissions represent around 30% of total hospitalizations. The remaining 70% are likely associated with comorbidities arising over time as the disease’s natural course, contributing to adult admissions in departments such as neurology, oncology, nephrology, and psychiatry. Therefore, early recognition of porphyria—particularly in commonly involved outpatient and inpatient specialties (Table 2)—is essential for timely diagnosis, long-term follow-up, and appropriate management, ultimately improving patients’ quality of life.

The mean age at validation in this study was 35.12 years (Table 1), which is similar to that reported in Sweden (36 years) [6] and the USA (33 years in AIP patients) [19]. In contrast, a Japanese study reported a higher mean age at diagnosis for AHP patients (44.4 years) [4]. However, this age range in the Japanese study is generally consistent with findings from Bissell’s study [1].

In this study, porphyria predominantly affected females, which is consistent with previous reports [1, 19]. According to a review article, the most common type of porphyria is AIP, which has a 90% female predominance [1]. An observational study of 108 porphyria patients in the USA revealed that 81% were female [19]. However, some studies revealed a nearly equal sex distribution [4, 6]. A study of 1,244 porphyria patients in Sweden conducted from 1987 to 2015 revealed that 53% of the patients were female [6]. Another observational study in Japan, which included 391 patients with porphyria between 2008 and 2020, revealed that 54.7% of the patients were female [4]. This may be due to the influence of female hormones on acute attacks. Estradiol, a key female sex hormone, is known to induce heme synthesis, increasing the risk of acute episodes [20]. Pregnancy [20] and exogenous hormones, such as contraceptives [21], have also been linked to acute attacks. Although porphyria can manifest in childhood, most patients develop symptoms after puberty [22]. A review reported that the age of symptom onset was approximately 18–45 years in AIP patients [1]. Bonkovsky et al. reported that the average delay in the diagnosis of acute porphyria in the USA is 15 years [19]. Given that symptoms typically appear in individuals from their teens to their twenties, this delay may contribute to the predominance of AIP diagnoses in females and the typical diagnosis occurring in their 30s.

Porphyria patients demonstrate significantly greater healthcare utilization than does the general population. Between 1998 and 2019, the average number of outpatient visits per person per year in the general population of Taiwan ranged from 13.8 to 16.2 [23], whereas a cross-sectional study in 2010 reported an average of 12.2 outpatient visits per person per year [24]. Hospital admissions averaged 11.1–14.6 per 100 people annually from 1998–2019 [23], with the 2010 study reporting 0.13 admissions per person per year [24]. The number of emergency room visits averaged 0.22 per person per year in general population in the 2010 study [24].

Compared with the general population, the porphyria patients in this study presented significantly greater healthcare demands, including outpatient visits, emergency visits, and hospitalizations (Supplemental Table S3). A German cohort study similarly reported an increased disease burden in acute porphyria patients, with a median of 23.0 healthcare visits per patient (IQR: 13.0–38.0) in the first year compared with 16.0 visits per year in the general population. Hospitalization rates for patients with porphyria range from 27.4% to 33.8%, which is significantly higher than the 19.3% reported in the general population [9].

On the other hand, over the past 20 years, the cumulative increase in porphyria cases (Supplemental Table S1) has contributed to increasing trends in annual outpatient visits, emergency visits, and hospitalizations (Supplemental Table S3). Additionally, the average hospital stay increased from 13.53 days in 2001 to 33.43 days in 2022 (Supplemental Table S3). These factors impose a considerable healthcare burden and negatively impact patients’ quality of life.

The mortality rate of the porphyria patients in this study was 1543.4 per 100,000 individuals (Table 1), which was markedly higher than the average mortality rate of 672.6 per 100,000 individuals in the general population from 2002–2022 [25]. AHP patients had a 1.3-fold greater risk of premature death than did the general population in the Sweden cohort study [26]. The average age of death for patients with porphyria was 62.38 years, whereas the average age of death for the general population in Taiwan in 2022 was 79.84 years [27]. This is consistent with findings from a long-term follow-up study conducted at Helsinki University Hospital, which reported a median age at death of 65 years, with a range of 19–87 years [28]. The German epidemiological study provides limited data on mortality in patients with porphyria [9]. In contrast, this study offers a comprehensive assessment of mortality, contributing to a more complete understanding of the disease’s natural history. The Porphyria patients in this study were relatively young and predominantly female, both of which are typically considered favorable factors for longevity. However, the findings revealed a higher mortality rate and earlier age at death among patients with porphyria. This suggests that the true burden of the disease may be greater than the statistics alone indicate.

In the past, treatment options for patients with AHP were limited. Early diagnosis is crucial for symptom prevention, with strategies including avoidance of trigger factors, ovulation suppression, hemin prophylaxis [1, 29] and heme arginate prophylaxis [13]. During acute attacks, management options include carbohydrate loading and hemin administration [30]. In AHP, hepatic delta-aminolevulinic acid synthase 1 upregulation leads to the accumulation of ALA and PBG, contributing to both acute attacks and chronic symptoms. Recently, givosiran, an RNA interference therapy, was shown to inhibit hepatic delta-aminolevulinic acid synthase 1 expression [29] and has been approved in multiple countries for AHP management [30]. Clinical studies have demonstrated its effectiveness in reducing both attack frequency [29] and severity [17]. Early diagnosis allows patients to receive appropriate treatment, potentially improving quality of life and alleviating healthcare burdens. Future therapies, including givosiran, may further extend lifespan. Additionally, as seen in advancements in the field of spinal muscular atrophy [31], the development of neonatal screening for AHP could facilitate earlier detection and enhance patient outcomes in the future.

### Limitations

First, patients who were potentially diagnosed with porphyria but had not yet obtained a catastrophic illness card may have been excluded, potentially leading to an underestimation of the true prevalence and incidence. However, this situation affects only the short-term incidence due to delayed diagnosis. Given that this study spans 20 years, its impact on long-term prevalence is likely limited. Second, Catastrophic Illness certification supports diagnostic rigor for porphyria but does not capture subtype information. In addition, ICD-9 and ICD-10 codes lack sufficient specificity to classify subtypes in this study. Consequently, we report aggregate outcomes for the overall cohort and acknowledge that unmeasured subtype heterogeneity could underlie differences in complications and outcomes. Outpatient and inpatient data based on ICD-10 codes (Tables 3 and 4) suggest that AIP is the most common subtype in Taiwan, which is consistent with the findings of previous studies [1, 4, 8, 19] in other countries. Additionally, a study from database identified AIP as the predominant subtype among the Chinese population [32]. Therefore, despite a substantial proportion of cases in outpatient ICD-10 records (Table 3) being classified as unspecified porphyria or other porphyria—collectively accounting for nearly half of porphyria-related ICD-10 codes—it is reasonable to infer that AIP remains the predominant subtype within this study. Moreover, real-world data from Japan suggest that patients with AIP had higher attack rates and more episodes in absolute terms than other AHP subtypes [4]. In this context, givosiran offers cautious optimism for fewer long-term complications and improved prognosis. However, future studies with prolonged follow-up are needed both to confirm these effects and to more precisely define subtype distribution. Third, because the dataset applied for this study lack pedigree information and do not release individual genetic test results, this study could not assess familial aggregation or distinguish sporadic from familial cases; genotype-level information was also unavailable. Finally, as this study focused on the Taiwanese population, the findings may not be generalizable to other ethnic groups. Nevertheless, this territory-wide, population-based, long-term study provides valuable epidemiological insights into porphyria, offering a foundation for future research and healthcare planning.

## Conclusion

The incidence and prevalence rates of porphyria in patients in this territory-wide, population-based study are lower than those reported in other countries. This study identified AIP as the most common subtype in Taiwan, which is consistent with previous research. Given the nonspecific symptoms and relatively young age of onset, diagnosing this rare disease remains challenging. Porphyria patients have significantly higher rates of outpatient visits, emergency visits, hospital admissions, and mortality than the general population does, highlighting both a reduced quality of life and a substantial burden on the healthcare system. Given the availability of new and effective treatments for AIP, early diagnosis and timely referral are crucial. Primary care providers, including family medicine physicians and general practitioners, play a key role in early recognition. Increasing awareness could improve patient outcomes while reducing the strain on healthcare resources.

## Supplementary Information

Below is the link to the electronic supplementary material.

Supplementary Material 1

## Data Availability

All the data presented in the manuscript can be found in Taiwan’s National Health Insurance Research Database (NHIRD).

## Abbreviations

AHP: Acute hepatic porphyria
AIP: Acute intermittent porphyria
RD: Rare disease
NHIRD: National Health Insurance Research Database
NHI: National Health Insurance
PBG: Porphobilinogen
ALA: Delta-aminolevulinic acid
ICD-9/10-CM: International Classification of Diseases, 9th/10th Revision, Clinical Modification
