# Supplementary material for: Epidemiological characteristics and natural history of porphyria – a twenty-year population-based analysis in Taiwan
Source: Orphanet J Rare Dis. 2025 Nov 17;20:586. doi: 10.1186/s13023-025-04110-7 (PMC12625002; doi:10.1186/s13023-025-04110-7)
Supplement: Supplementary file 1 — Supplementary Material 1 [file 13023_2025_4110_MOESM1_ESM.docx]

Supplemental Table S1. The incidence, prevalence and mortality rates of porphyria patients in Taiwan between 2002 and 2022

| Year | Incidence  (per million) | Prevalence  (per million) | | | Mortality rate  (per 10,000) |
| --- | --- | --- | --- | --- | --- |
| 2002 | 0.04 | | 0.04 | 0.0 | |
| 2003 | 0.22 | | 0.27 | 0.0 | |
| 2004 | 0.53 | | 0.79 | 0.0 | |
| 2005 | 0.62 | | 1.41 | 0.0 | |
| 2006 | 0.35 | | 1.75 | 0.0 | |
| 2007 | 0.09 | | 1.83 | 0.0 | |
| 2008 | 0.48 | | 2.30 | 0.0 | |
| 2009 | 0.17 | | 2.47 | 1754.4 | |
| 2010 | 0.17 | | 2.59 | 0.0 | |
| 2011 | 0.17 | | 2.76 | 0.0 | |
| 2012 | 0.34 | | 3.09 | 1388.9 | |
| 2013 | 0.13 | | 3.17 | 0.0 | |
| 2014 | 0.34 | | 3.50 | 2439.0 | |
| 2015 | 0.21 | | 3.62 | 0.0 | |
| 2016 | 0.21 | | 3.83 | 0.0 | |
| 2017 | 0.30 | | 4.12 | 1030.9 | |
| 2018 | 0.25 | | 4.33 | 980.4 | |
| 2019 | 0.13 | | 4.41 | 0.0 | |
| 2020 | 0.25 | | 4.66 | 0.0 | |
| 2021 | 0.30 | | 4.99 | 0.0 | |
| 2022 | 0.13 | | 5.15 | 1666.7 | |
| Average | 5.44 † | | 5.18 ‡ | 1543.4 § | |

† Average incidence is calculated as the total number of newly diagnosed cases from 2002--2022 divided by the average mid-year population over the same period.

‡ Average prevalence is defined as the total number of cases in 2022 divided by the average mid-year population from 2002--2022.

§ Years with zero deaths were excluded from the calculation.

Supplemental Table S2. List of ICD-9 and ICD-10 Codes with Diagnoses Related to This Study

| **ICD9** | | **ICD10** | |
| --- | --- | --- | --- |
| 277.1 | Disorders of porphyrin metabolism | A419 | Sepsis, unspecified organism |
| 295.34 | Schizophrenic disorders, paranoid type, chronic with acute exacerbation | C229 | Malignant neoplasm of liver, not specified as primary or secondary |
| 295.80 | Other specified types of schizophrenia, unspecified | D500 | Iron deficiency anemia secondary to blood loss (chronic) |
| 401.9 | Essential hypertension, unspecified | D6861 | Antiphospholipid syndrome |
| 460 | Acute nasopharyngitis (common cold) | E800 | Hereditary erythropoietic porphyria |
| 461.9 | Acute sinusitis, unspecified | E801 | Porphyria cutanea tarda |
| 462 | Acute pharyngitis | E8020 | Unspecified porphyria |
| 463 | Acute tonsillitis | E8021 | Acute intermittent (hepatic) porphyria |
| 465.9 | Acute upper respiratory infections of unspecified site | E8029 | Other porphyria |
| 466.0 | Acute bronchitis | F03 | Unspecified dementia |
| 558.9 | Other and unspecified noninfectious gastroenteritis and colitis | F200 | Paranoid schizophrenia |
| 560.1 | Paralytic ileus | F341 | Dysthymic disorder |
| 560.81 | Intestinal or peritoneal adhesions with obstruction(postoperative)(postinfection) | I10 | Essential (primary) hypertension |
| 599.0 | Urinary tract infection, site not specified | I629 | Nontraumatic intracranial hemorrhage, unspecified |
| 692.9 | Unspecified contact dermatitis, unspecified cause | I679 | Cerebrovascular disease, unspecified |
| 706.1 | Other acne | J069 | Acute upper respiratory infection, unspecified |
| 7109 | Unspecified diffuse connective tissue disease | J189 | Pneumonia, unspecified organism |
| 789.00 | Abdominal pain, unspecified site | K559 | Vascular disorder of intestine, unspecified |
|  |  | L039 | Cellulitis and acute lymphangitis, unspecified |
|  |  | N186 | End stage renal disease |
|  |  | N390 | Urinary tract infection, site not specified |
|  |  | R109 | Unspecified abdominal pain |

Supplemental Table S3. Average annual healthcare utilization per person among patients with porphyria between 2001 and 2022

| Year | Outpatient Visits  (times) | Emergency Visits (times) | | | Inpatient Admission (times) | LOS in ACB† (days) |
| --- | --- | --- | --- | --- | --- | --- |
| 2001 | 15.99 | | 2.85 | 2.06 | | 13.53 |
| 2002 | 16.56 | | 3.46 | 2.71 | | 19.38 |
| 2003 | 16.44 | | 2.98 | 2.83 | | 19.29 |
| 2004 | 17.37 | | 3.33 | 2.63 | | 22.75 |
| 2005 | 18.91 | | 4.22 | 3.23 | | 37.32 |
| 2006 | 20.23 | | 5.75 | 3.00 | | 20.42 |
| 2007 | 21.69 | | 5.20 | 2.37 | | 21.40 |
| 2008 | 20.60 | | 4.80 | 3.26 | | 28.24 |
| 2009 | 23.31 | | 5.22 | 3.52 | | 28.48 |
| 2010 | 20.85 | | 5.25 | 3.82 | | 22.71 |
| 2011 | 23.88 | | 4.91 | 3.55 | | 27.58 |
| 2012 | 25.30 | | 6.28 | 4.36 | | 25.18 |
| 2013 | 25.38 | | 6.63 | 4.38 | | 40.07 |
| 2014 | 27.19 | | 6.70 | 4.35 | | 36.35 |
| 2015 | 28.06 | | 7.03 | 4.25 | | 24.78 |
| 2016 | 29.61 | | 7.85 | 5.87 | | 45.67 |
| 2017 | 31.09 | | 9.11 | 6.58 | | 48.32 |
| 2018 | 31.34 | | 8.03 | 4.98 | | 32.49 |
| 2019 | 30.28 | | 8.20 | 4.65 | | 55.31 |
| 2020 | 31.32 | | 6.65 | 4.18 | | 24.69 |
| 2021 | 34.20 | | 7.09 | 4.88 | | 28.05 |
| 2022 | 35.54 | | 7.43 | 4.60 | | 33.43 |

† LOS in ACB: length of stay in acute care bed
